# The Role of Zinc Finger Proteins in Various Oral Conditions

**DOI:** 10.1155/2022/4612054

**Published:** 2022-04-15

**Authors:** Rupali Agnihotri, Sumit Gaur

**Affiliations:** ^1^Department of Periodontology, Manipal College of Dental Sciences, Manipal Academy of Higher Education (MAHE), Manipal 576104, Karnataka, India; ^2^Department of Pedodontics and Preventive Dentistry, Manipal College of Dental Sciences, Manipal Academy of Higher Education (MAHE), Manipal 576104, Karnataka, India

## Abstract

The zinc finger proteins (ZNFs) are essential transcription factors, and the genes encoding them constitute about 3% of the entire human genome. They are involved in the development of several tissues, and any alterations in their structure may promote chronic conditions like diabetes and tumorigenesis. Lately, their role in the development, progression, and metastasis of Oral Squamous Cell Carcinoma (OSCC), Epithelial Dysplasia, Oral Lichen Planus, and Periodontitis has been found. The present review aims to describe their role in various oral conditions. Electronic databases like Medline (PubMed) and Scopus were searched for original studies related to the role of ZNFs in various oral conditions. It yielded 48 studies included in the review. It was found that the ZNFs influenced chronic conditions like Oral Cancer and Periodontitis. They act both as tumor suppressors and oncogenes and have an anti-inflammatory effect. The knowledge from the present review may be utilized in designing drugs that prevent unusual expression of specific ZNFs. Besides, they may be applied as prognostic markers due to their high expression specificity in some tumors.

## 1. Introduction

The zinc finger proteins (ZNFs) are important transcription factors, and their encoding genes constitute about 3% of the human genome [1, 2]. Initially discovered in the 1980s as the DNA-binding motifs in the nuclei of cells from the African clawed frog, *Xenopus laevis*, they are one of the largest groups of proteins [1, 3]. They include various zinc finger domains that facilitate interactions with RNA, DNA, poly-ADP-ribose, and other proteins [4]. Besides, they regulate several cellular processes like ubiquitin-mediated protein degradation, transcription, signal transduction, actin targeting, cell migration, and DNA repair [4].

**Figure 1:**
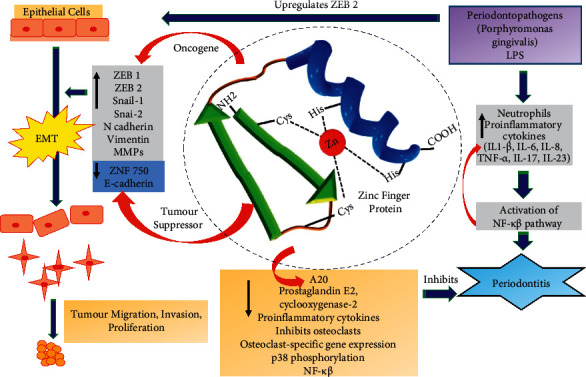
Zinc finger proteins in oral cancer and periodontitis.

The ZNFs are unique as the zinc ions form a complex with two cysteine and two histidine residues (C2H2) in a peptide sequence (Figure 1) [4, 5]. It results in a finger-like, three-dimensional configuration consisting of two *β*-sheets and one *α*-helix [4, 5]. There are about 30 different types of ZNFs based on their domain structure [4, 6]. Some of them include C2H2, really interesting new gene (RING), plant homeodomain (PHD), and Lin-ll, Isl-1, and Mec-3 (LIM domains) [4].

The ZNFs are involved in the development of several tissues, and any alterations in their structure may result in chronic conditions like neurodegenerative disorders, diabetes, and tumorigenesis [4]. Lately, their role in the progression and metastasis of oral squamous cell carcinoma (OSCC) and periodontitis has been identified [7–9].

About 90% of the oral cancers in the world are OSCC managed by either surgery, radiation, or coadjutant therapy [10]. They have a poor prognosis with a high mortality rate [10, 11]. Early diagnosis and treatment are the basis for improving their survival, and ZNFs are vital in this aspect. They may act as oncogenes or tumor suppressors as they influence protein transcription. They affect all cancer progression pathways by recruiting chromatin modifiers or structural proteins that control cancer cell migration and invasion [4]. Some examples of the oncogenic ZNFs include ZNF281, ZBP89, Mouse Double Minute 2 (MDM2), and zinc finger E-box-binding homeobox proteins (ZEB)1 and 2 [4]. The tumor suppressor ZNFs include ZNF750, ZNF185, and zinc fingers and homeoboxes-1 (ZHX1) [4]. They trigger cell apoptosis, inhibit ribosome synthesis, and nuclear factor kappa B (NF-*κ*B) and Activator protein-1 (AP-1) signaling [2]. The ZNFs specifically identified in OSCC are ZNF750, ZNF582, Glioma-Associated Oncogene (Gli) family, Snail1, Slug, A20, Krüppel-like factors (KLF), ZEB1, and ZEB2 [12–35].

The ZNFs may be important therapeutic targets in periodontitis [9, 36–39]. For instance, overexpression of the ZNF A20 inhibits activation of NF-*κ*B in periodontitis. Besides, it blocks osteoclastic differentiation. The keystone periodontopathogen, *Porphyromonas gingivalis* (*P. gingivalis*), promotes tumorigenesis by disrupting epithelial cell pathways. Additionally, it stimulates ZEB1 expression in immortalized gingival epithelial cells along with Snail Family Transcriptional Repressors (SNAI) 1and 2. They promote a mechanistic convergence between the tumorigenic potential and periodontal diseases owing to their ability to control inflammatory responses [37, 38]. The *P. gingivalis* hinders apoptosis and increases the cell cycle progression. It disrupts local inflammatory responses and increases the epithelial-mesenchymal transition (EMT). As a result, the epithelial cells transform and develop a motile phenotype, producing self-renewing tumor-initiating cells. In malignant tumors, transcription factors like ZEB1 and ZEB2, SNAI1 and SNAI2, and Twist-related protein (TWIST) 1 and 2 may lead to migratory and invasive cancer cells [37]. In this context, the present review aims to describe the role of ZNFs in various oral conditions.

## 2. Materials and Methods

**Figure 2:**
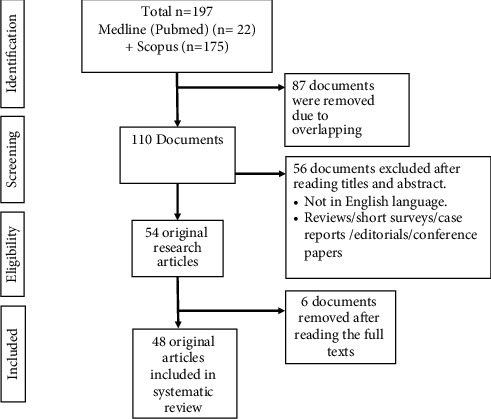
PRISMA flow for literature search.

The research publications on the influence of ZNFs in various oral conditions were identified with the help of the Preferred Reporting Items for Systematic Reviews and Meta-analyses (PRISMA) guidelines. “Zinc finger protein” And “Oral” AND “Disease” OR “Oral squamous cell carcinoma” OR “Oral cancer” OR “Periodontitis” were the keywords applied in the databases like Medline (PubMed) and Scopus. The initial search leads to 197 articles (Figure 2). After screening for duplicates, the titles and abstracts of 110 articles were read. Original full-text articles in English related to the role of ZNFs in oral conditions were included, while recommendations, reviews, technical reports, case reports, and expert statements were excluded.

In total, 54 original research articles were read, and six articles were omitted. Finally, 48 original studies were included [7, 9, 12–57]. Their aims and objectives, type of ZNF, the oral condition, and the role of ZNF in the condition were recorded (Table 1).

## 3. Results and Discussion

Among the 48 studies, twenty-two studies reported results from the tissue samples obtained from human subjects [7, 9, 16, 17, 20, 22, 25, 27, 30, 32, 35, 36, 41–43, 46, 49, 50, 52, 53, 56, 57], while two studies involved animal models [21, 33]. The following sections discuss different ZNFs in various oral conditions like OSCC [7, 12–20, 22–27, 29–33, 35, 40, 41, 43, 45–53], nasopharyngeal carcinoma [28], salivary adenoid cystic carcinoma [42], oral verrucous carcinoma [44], lichen planus [54], epithelial dysplasia [16, 40], and periodontitis [9, 21, 36–39, 56, 57].

### 3.1. ZNFs Reported in Various Studies

The various ZNFs reported in the included studies were Gli, ZEB1, ZEB2, ZNF582, zinc finger associated with squamous cell carcinoma (ZASC1), repressor element-1 silencing transcription factor (REST), A20, KLF, gut-enriched Krüppel-like factor (GKLF), ZNF185, Snail, Slug or SNAI2, Myeloid zinc finger 1 (MZF1), Krüppel-like zinc finger transcription factor 9 (ZF9), zinc finger AN1-type containing-4 (ZFAND4), ZNF750, Zinc finger protein X-linked (ZFX), Keratin 17 (KRT17), interferon regulatory factor (IRF), PR domain zinc finger protein1 (PRDM1), nucleus accumbens-associated protein 1 (NAC1), zinc finger and BTB domain-containing 7A protein (ZBTB7A), monocytic leukemia zinc finger protein (MOZ-) related factor (MORF), ZNF718, Homeobox A4 (HOXA4), and ZNF57 [7, 9, 12–57].

The ZEB1 and ZEB2 [25, 26, 31–35, 37, 38, 49, 54] were the most studied ZNFs followed by Gli [19–22, 51], ZNF750 [12–15], Snail [23, 25, 26, 29], Slug [24, 25, 42], ZNF582 [16–18], A20 [9, 28], KLF [29, 30, 43], and ZFAND4 [50, 52]. Other ZNFs like ZASC1 [43], REST [45], GKLF [40], ZNF185 [41], MZF1 [46], ZF9 [36], ZFX [47], KRT17 [22], IRF [39], PRDM1 [39], NAC1 [48], ZBTB7A [55], MORF [56], ZNF718, HOXA4, and ZNF57 [57] were evaluated in very few studies.

### 3.2. Oral Conditions Influenced ZNFs

The role of ZNFs was primarily observed in the progression and inhibition of OSCC, followed by other oral conditions like lichen planus, epithelial dysplasia, and periodontitis [7, 9, 12–17, 19–33, 35–54]. Some studies investigated the influence of the periodontopathogen P gingivalis on ZNFs and their subsequent role in the development of OSCC [25, 33]. A few studies reported the effects of microRNAs (miR) on ZNFs and the progression of OSCC and periodontitis [31–34, 38, 49, 55–57].

#### 3.2.1. ZNFs Involved in Oral Cancer, Lichen Planus, and Epithelial Dysplasia

The ZNFs of the C2H2 domain play an essential role in cancer progression by controlling the transcription of downstream genes concerned with proliferation, apoptosis, migration, and invasion [2]. Environmental stimuli that activate signaling cascades and improve ZNF functions through posttranslational modifications (e.g., phosphorylation and acetylation) and various cancer-related miRs (miR-199a-3p, miR-525-3p, miR-940, and miR-31) regulate tumorigenesis. They control their DNA binding ability and recruitment of interacting proteins, transcription coactivators/corepressors, chromatin modifiers, and other transcription factors [2].

Evidence shows that ZNFs are involved in oral cancer progression [2, 35]. EMT, which involves altering epithelial cells into mesenchymal cells with simultaneous changes in the cell form, morphology, and adhesion, plays a crucial role in cancer development and metastasis [35, 58–60]. In this process, the expression of E-cadherin, a vital adhesion molecule for maintaining epithelial integrity, is lost [61]. Besides, mesenchymal molecules such as N-cadherin and vimentin are overexpressed [62]. However, they promote tumor migration and invasion [35]. The E-cadherin expression is controlled by various transcription factors like ZEB1, ZEB2, and Snail, which downregulate it by particularly binding to the E-boxes domain and then inducing the EMT (Figure 2) [35].

*(1) ZEB-1*. ZEB-1, a transcription factor, is significant for the invasion and metastasis of various human cancers, including oral cancer [35, 63]. It induces EMT by controlling the target genes through protein binding domains, including the Smad, C-terminal binding protein (CtBP), and p300-P/CAF domains [35, 64, 65]. It binds its E-box-like sequences within the E-cadherin promoter region with the help of two zinc finger domains at the N- and C-termini, which enables specific regulation of E-cadherin expression [35, 66]. In OSCC, reduced E-cadherin expression is correlated with loss of epithelioid cell morphology, metastasis, and poor prognosis [35, 67].

The overexpression of miR-429 inhibited OSCC cell lines growth and vice versa due to inhibition of ZEB1 expression; i.e., the two were negatively correlated [31]. Furthermore, the periodontopathogen, *P gingivalis*, increases the ZEB1 expression as a dual-species community with *Fusobacterium nucleatum* (*F. nucleatum*) or *Streptococcus gordonii* (*S. gordonii*) [33]. However, its strains lacking the FimA fimbrial protein did not induce ZEB1 expression. Its expression was positively correlated with mesenchymal markers like the vimentin and matrix metalloproteinase-9 (MMP-9). The knockdown of ZEB1 inhibited their increase and cell migration caused by the *P. gingivalis* [33]. Even in the mice, the *P.* gingivalis increased ZEB1 levels in gingival tissues samples from OSCC [33]. Therefore, the FimA-driven ZEB1 expression could provide a mechanistic basis for the role of *P. gingivalis* in OSCC [33].

In OSCC, the ZEB1 expression, metastasis, and poor prognosis were inversely related to the downregulation of miR-101, which inhibits cancer cell proliferation, apoptosis resistance, migration, and invasion in vitro. As miR-101 directly affects the ZEB1, it could be a potential therapeutic target for OSCC [49].

The ZEB-1 and the E-cadherin expression were inversely related [35]. They were associated with tumor recurrence, metastasis, and pathologic grading. ZEB-1 positivity with loss of E-cadherin expression leads to a poor prognosis. Higher ZEB-1 and lower E-cadherin mRNA expression were observed in OSCC. The ZEB-1 expression was regarded as a potential prognostic marker of OSCC [35].

It was also evaluated in oral lichen planus, but its role was unclear in the condition [54].

*(2) ZEB2*. The ZEB2, or Smad Interacting Protein 1 (SIP1), is a transcription factor and a protein related to the transforming growth factor-*β* (TGF-*β*) signaling cascade [68]. It interacts with Smad, binds to DNA, and acts like a multizinc finger transcription factor involved in multiple cellular functions [69]. It is a transcriptional inhibitor of E-cadherin and is involved in the EMT in various types of carcinomas [68]. Further, it promotes tumor angiogenesis, which is significant for cancer cell unrestrained growth and metastasis [70].

In OSCC, a direct interaction between miR-200b, ZEB2, and Kindlin-2 mRNA was observed [32]. The expression levels of Kindlin-2 and ZEB2 were significantly elevated, while those of miR-200b mRNA were downregulated in OSCC cells [32]. While the miR-200b directly targeted ZEB2 and repressed both the migration and invasive functionality of the cancer cell lines, both Kindlin-2 and ZEB2 accelerated their migration and invasion [32]. It was found that Kindlin-2 controlled ZEB2 expression independent of miRNAs [32].

Besides, a study showed that in OSCC, the miR-345 induced cell cycle arrest in the G1 phase [34]. It reduced both mRNA and protein expression of ZEB2. Because it can target ZEB2, miR-345 could be applied as a tumor inhibitor in OSCC treatment [34].

*(3) Gli1 and Gli2*. The Gli-ZNF, a glioma-associated oncogene, is a protein encoded by the Gli-1 gene in humans [71]. The Gli family ZNF (Gli-1, Gli-2, and Gli-3) are downstream signaling factors for the Sonic hedgehog (SHH) pathway [71]. They are essential for controlling developmental processes. Furthermore, the SHH/Gli's promote tumor cell growth.

Evaluation of the role of regulators of Gli2 (Hedgehog, TGF-*β*, and Wnt signaling) in controlling the parathyroid hormone expression (PTH) suggested that the canonical Hedgehog and TGF-*β* signaling increased the PTH expression and mandibular invasion in a Gli2-dependent manner [19]. Moreover, inhibition of Gli2 significantly decreased both PTH expression and bony invasion. As multiple signaling pathways converged on Gli2 to mediate this process, it was suggested that Gli2 could be a therapeutic target to prevent bony invasion in OSCC [19]. Furthermore, increased SHH and Gli1 were reported in OSCC [49]. The SHH expression positively correlated with the microvessel density, TNM stage, tumor recurrence, and lymph node metastasis.

Furthermore, Gli1 or Gli2 were frequently detected in KRT17 positive regions of OSCC [22]. Here they promoted tumor cell growth through their antiapoptotic effect. The knockdown of Gli3 in OSCC was associated with a significant decrease in different cancer stem cell-like fractions, spheres and colonies, downregulation of the CD44, octamer-binding transcription factor-4, and BMI1 genes with an increase in the expression of the involucrin and S100A9 genes [51]. It inhibited cellular proliferation and invasion. Although a high Gli3 expression was associated with tumor size, it did not determine the prognosis. However, it contributed to the OSCC stemness and malignant behavior [51].

*(4) ZNF 750*. The ZNF750 is a lineage-specific tumor suppressor gene related to squamous cell carcinoma (SCC) [72]. It is a transcriptional regulator of epidermal differentiation, controlling epidermal progenitor genes and inducing their differentiation. As it is a tumor suppressor gene, its overexpression may inhibit carcinomatous cell proliferation, invasion, and migration.

An RNA sequence profiling for the genes and pathways involved in tumor suppression by overexpression of ZNF750 in OSCC showed augmentation of cell cycle-associated genes, which stimulated cell cycle arrest in the G0/G1 phase, a critical factor in the antitumor effect on OSCC cells [12]. Another study revealed that overexpression of ZNF 750 altered the mRNA expression profiles in OSCC and affected the genes related to oxidative stress, Wnt, Janus kinase/signal transducers and activators of transcription (JAK/STAT), TGF-*β*, NF-*κ*B, p53, Notch, Hedgehog, Peroxisome Proliferator activated receptor (PPAR), and hypoxia signaling [12]. It was believed that the ZNF750 regulated various signaling pathways in CAL-27 cell lines [12]. It suppressed the malignancy due to its ability to inhibit the protein or mRNA expression of angiogenin, vascular endothelial growth factor, G protein signal-regulated protein 5 (RGS5) and CD105, repression of cell adhesion molecules, and upregulation of the protein or mRNA expression of prolyl hydroxylase 2 and platelet-derived growth factor-*β* [14]. As ZNF750 altered the tumor vascular microenvironment, it further inhibited the malignant progression of OSCC. It even decreased the expression of MMP-28, cyclin B1, and the mesenchymal marker, neural cadherin [15]. It induced the differentiation-associated genes and triggered the expression of the late epidermal differentiation factor, the KLF-4 [15]. Finally, in CAL-27 cell lines, it inhibited cell invasion migration and prevented metastasis of OSCC [15].

*(5) ZNF 582*. The ZNF 582 is located at chromosome 19q13.43 and contains one Krüppel-associated box (KRAB) AB domain and nine zinc finger motifs [71, 73]. Together with the paired-box1 (PAX1) gene located on chromosome 20p11.2 p, it plays a significant role in oral cancer [74]. The PAX1 and ZNF582 hypermethylation was found in oral and cervical cancer lesions scrapings and associated with malignant progression and poor prognosis [17]. In primary and recurrent OSCC, hypermethylation of PAX1 or ZNF582 was observed [17]. The promoter methylation was observed in stage III or IV and bone invasion cases in the primary sites. A sudden increase in methylated ZNF582 and PAX1 from mild to moderate or moderate or severe dysplasia suggests that hypermethylated PAX1 and ZNF582 could serve as biomarkers for the severity of OSCC [16]. Furthermore, the higher M-index of methylated ZNF582 and PAX1 was significantly correlated with more advanced tumor and shorter survival rates [17].

*(6) Snail1 and Snai2 (Slug)*. Snail, encoded by the SNAI1 gene, and Slug, encoded by the SNAI2 gene, are ZNFs belonging to the Snail family. They are expressed in the skeletal stem or stromal cells during the pre- and postnatal states [75]. During the EMT, the Snail and Slug inhibit E-cadherin transcription. They are abnormally expressed in some cancers and regulate cell proliferation, apoptosis, and motility [75].

In OSCCs, the cells from primary carcinoma showed the phenotype of squamous epithelial cells, including E-cadherin and laminin-332 (laminin-5), while those from the recurrent tumors exhibited characteristics of dedifferentiated tumors with EMT [26]. The Snail-transfected cells exhibited a complete EMT phenotype with fibroblastoid appearance, vimentin filaments, E and N-cadherin switch, lack of hemidesmosomes, and laminin-332 synthesis [26]. Additionally, the ZEB-1 and ZEB-2 were upregulated, suggesting that Snail regulated the E-cadherin inhibitors.

Further, a direct interaction between DNA binding protein inhibitor-ID2 and Snail1 was found [23]. The ID2 expression triggered a malignant phenotype with invasive properties through the ID2–Snail axis and was suggested as a potential therapeutic target for OSCC [23].

The Slug expression also modifies the adherens and desmosomal junctions. Long-term P gingivalis infection may increase Slug levels and other EMT-associated transcription factors, like Snail and ZEB-1, due to its ability to promote EMT phenotype and cell migration, which was slightly enhanced in cases of coinfection with *F. nucleatum* [25]. Besides, a positive Slug expression was reported in the saliva of patients with salivary adenoid cystic carcinoma [42].

*(7) A20*. A20 was initially recognized as a tumor necrosis factor- (TNF-) *α* induced primary response gene in human umbilical vein endothelial cells, which encodes a novel ZNF [76]. Several stimuli induced A20 in various cell types like fibroblasts, breast carcinoma cell lines, Jurkat T cells, and U937 promonocytic cells. It inhibits apoptosis [76].

The A20 RNA was expressed in 76% of undifferentiated nasopharyngeal carcinoma and 80% of poorly differentiated SCC [28]. However, it was not detected in well-differentiated SCCs of the skin or any normal samples.

*(8) KLF*. The KLF family comprises diverse homologous genes that function as DNA-binding transcriptional regulators, which control cell proliferation, differentiation, and migration and maintain pluripotency [77]. They have triple zinc finger DNA-binding domains at the carboxyl terminus, but other regions are highly divergent [77]. An activation or repression domain is located at the amino terminus, and alternative splicing of some KLFs can lead to additional alterations in protein structure [77]. The KLFs act as tumor suppressors or oncogenes. The KLF4 and KLF7 were evaluated in two included studies [29, 30].

The KLF4 is a zinc finger transcriptional factor highly expressed in differentiated, postmitotic cells in both gut and skin epithelium and the lung, testis, thymus, cornea, cardiac myocytes, and lymphocytes [30]. As it has both activation and repressor domains, it exerts a positive or negative transcriptional effect on target tissues depending on the type of tissue [30]. It regulates cellular proliferation, differentiation, migration, and apoptosis and maintains normal tissue homeostasis as a transcription factor.

However, it may act as an oncogene in some specific cancers. For instance, its expression was significantly decreased in oral cancer [30]. Its suppression was related to the KLF4 promoter hypermethylation with reduced expression in poorly differentiated oral cancers. Even though it shows anticancer effects by inhibiting cell proliferation, cell cycle progression, colony formation, and apoptosis induction, its overexpression may cause cell migration and invasion. Its knockdown leads to cancer cell growth and colony formation with simultaneous cell migration and invasion inhibition. The MMP-9 may promote KLF4-mediated cell migration and invasion, resulting in its “Janus-faced” roles in oral carcinogenesis, acting both as a tumor suppressor and as an oncogene [30].

Besides KL4, the KLF7 promotes migration and EMT in human OSCC. Its overexpression changed their migratory behavior and triggered EMT and lymph node metastasis through the expression of Snail [29].

*(9) ZFAND4*. The zinc finger, ZFAND4 also known as AN1 ubiquitin-like homolog (ANUBL1), is one of the most upregulated genes identified in recurrent OSCC [52, 78]. Its expression levels are usually higher in advanced cases. The ZFAND4 promotes cell proliferation by activating cyclin-dependent kinase and downregulation of p21 and p53 [52, 79].

Increased cytoplasmic expression of ZFAND4 was observed in about 21% of the subjects with OSCC [50]. Its overexpression was considered an independent poor prognostic factor and marker for predicting metastasis [50]. Likewise, increased ZFAND4 staining in the undifferentiated areas of tumors in about 125 patients correlated with the tumor location [52]. Its increased expression was seen in well-differentiated and nonrecurrent tumors. However, no correlations were observed between the ZFAND4 expression and patient survival, and distinct ZFAND4 expression patterns had to be studied [52].

*(10) Other ZNFs*. Some other ZNFs that were evaluated in very few studies ZASC1 [43], REST [45], GKLF [40], ZNF185 [41], MZF1 [46], ZFX [47], KRT17 [22], IRF [39], PRDM1 [39], ZBTB7A [55], and NAC1 [48].

The ZASC1 is a ZNF transcription factor localized on chromosome 3q26, which carries oncogenes frequently altered in neoplasms [43]. An examination of twenty-seven OSCC patients with primary and recurrent tumors revealed that the ZASC1 copy number increased progressively from primary to recurrent tumors. It was associated with tumor progression and betel quid consumption in recurrent tumors [43]. Besides, the OSCC cells expressing ZASC1 showed increased proliferation, and its knockdown reduced the growth and colony formation of the cancer cells. It was also associated with the recurrence of OSCC.

Another ZNF evaluated was REST, the Neuron-restrictive silencer factor (NRSF). It is a major transcriptional repressor for neuron-specific genes in nonneuronal and neuronal progenitor cells. Higher expression of REST was found in the human OSCC-KB cell line, while its knockdown reduced the cell viability due to apoptosis and DNA fragmentation, suggestive of its contradictory roles in tumor suppression and cancer progression [45]. It even disrupted the mammalian target of the rapamycin (mTOR) signaling pathway, a critical survival factor in many types of cancer cells [45]. Another ZNF, the GKLF or epithelial zinc finger expression, was detected in the upper, differentiating cell layers of the oral squamous epithelium, specifically the dysplastic epithelium [40]. It was considered an oncogene that regulated the proliferation or differentiation in epithelia.

Some studies found the downregulation of a ZNF185 in head and neck cancers [41]. The MZF1 was expressed in 69.3% of patients with OSCC [46]. The loss of nuclear expression of MZF1 was seen in advanced tumors. In the tongue SCC, negative nuclear MZF1 expression worsened the survival rates [46]. Similarly, overexpression of ZFX led to development, while its knockdown suppressed tongue carcinoma [47].

Another protein, the NAC1, which controls several cellular functions, was considered a strong predictor of OSCC. It was also a potential marker for distinguishing oral epithelial dysplasia from OSCC [48].

#### 3.2.2. ZNFs Associated with Periodontitis

Chronic periodontitis (CP) is an immunoinflammatory disease triggered by the dental plaque biofilm that destroys tooth-supporting tissues leading to tooth loss [9, 80]. The lipopolysaccharides (LPS) of Gram-negative periodontopathogens like *P. gingivalis* stimulate destructive host responses, including leukocyte recruitment and proinflammatory cytokine (interleukin [IL]-1*β*, IL-6, IL-8, and TNF-*α*) release [9, 81, 82]. They trigger bone destruction and release of IL-17 and IL-23 from human periodontal ligament cells (HPDLCs) [9, 83]. Furthermore, the NF-*κβ* pathway enhances proinflammatory gene expression and destructive mediators release [9]. The ZNF A20 can inhibit NF-*κβ* pathway activation via inflammatory cytokine, toll-like, and NOD-2 receptors [9, 84]. Besides, A20 is activated by IL-1*β*, TNF-*α*, and LPS [9, 85–87]. Studies have revealed that its knockout produces severe inflammation, cachexia, and premature death, while its overexpression is neurotoxic [9, 88, 89].

The inhibition of A20 increased bone resorption in LPS-treated osteoclast cultures, and its overexpression was helpful in the management of inflammation and bone resorption in periodontitis [90]. Furthermore, it was upregulated in the gingival tissues and neutrophils from periodontitis subjects and in HPDLCs exposed to LPS and nicotine [9]. The ZNF-A20 reduced prostaglandin E2, cyclooxygenase-2, and proinflammatory cytokine release. It downregulated osteoclast-specific gene expression with reduced osteoclasts and inhibited the LPS or nicotine-induced p38 phosphorylation, NF-*κβ*, protein kinase C*α*, Akt, and GSK-3*β* pathways activation (Figure 1). Owing to these anti-inflammatory and anti-bone resorptive effects, A20 could be a potential therapeutic target in periodontitis [9].

Another ZNF, the ZF9, activates TGF-*β*. An analysis of the specific gene expression of neutrophils in generalized aggressive periodontitis and CP showed significantly higher mRNA levels of heat shock transcription factor 4b (HSF4b) gene, ZF9, and muskelin genes [36]. Even though HSF4b was increased, the ZF9 and muskelin genes were reduced in aggressive periodontitis compared to CP and healthy subjects. It was suggested that HSF4b, ZF9, and muskelin were transcription factors, activators of TGF-*β*, and influenced cellular adhesion, respectively. They altered neutrophil functions in aggressive periodontitis [36].

An animal study investigating the role of diabetes in periodontal disease and alveolar bone loss evaluated Gli homologs encoding Gli1, Gli2, and Gli3 zinc finger transcription factors [21]. The osteoblast differentiation was impaired in mice deficient in Gli1 and Gli3 or Gli1 and Gli2. It suggested that Gli collaborated with Gli2 and Gli3 during osteogenesis. Furthermore, the Hedgehog–Gli1 axis was indirectly involved in osteoclastogenesis. It was observed that hyperglycemia upregulated the osteoprotegerin and downregulated osteocalcin mRNA expression.

Further, the TNF-*α* mRNA expression was initially upregulated, followed by the alveolar bone loss at the third and seventh days in streptozocin-induced diabetic mice. The Gli1 and collagen type VI-*α*1 were downregulated in the gingiva of the ligated site with reduced alkaline phosphatase activity and enhanced levels of tartrate-resistant acid phosphatase positive multinucleated cells [21]. Another study identified IRF5 and PRDM1 as shared susceptibility factors between rheumatoid arthritis and aggressive periodontitis [39].

Polymicrobial communities influence the ZEB2, a transcription factor involved in EMT and inflammatory responses in periodontitis [37]. It was revealed that the *P. gingivalis* enhanced its expression via the pathways related to *β*-catenin and Forkhead Box O1 (FOXO1). However, *S. gordonii* antagonized ZEB2 expression by suppressing FOXO1 even in the presence of *P. gingivalis*. It was considered a homeostatic commensal, capable of modifying the activity of P gingivalis by altered host signaling.

Another ZNF, the MORF, was related to impaired function and prolonged stress of the endoplasmic reticulum and defective osteogenic differentiation of periodontal ligament stem cells [56].

Lately, it has been suggested that immune system modulation through gene regulation mechanisms plays a significant role in the progression and susceptibility of periodontitis. The epigenetic mechanisms like epigenetic DNA methylation, histone modification, and miRNA coordinate the gene regulation. Besides, DNA methylation in the blood helps detect treatment response predictors in several diseases, including periodontitis. In periodontitis, the DNA methylation patterns of ZNF718, HOXA4, and ZNF57 genes were observed in peripheral blood leukocytes. The ZNF718 and HOXA4 were hypermethylated, while ZNF57 was hypomethylated. The ZNF718 is associated with metabolic syndrome and diabetes, the HOXA4 acts as a transcription factor during embryogenesis and inhibits abnormal remodeling of vascular smooth muscle cells during inflammation, and ZNF57 is related to antigen presentation. Due to their immune regulatory and antigen processing functions, leukocyte DNA methylation could be applied to evaluate systemic immune-related epigenetic patterns in periodontitis [57].

#### 3.2.3. Plausible Applications of the Knowledge on ZNFs

The ZNFs are essential for tissue hemostasis and disease as they influence the recruitment of chromatin modifiers, cofactors, or structural proteins [4]. The ZNF may be applied in the management of various oral conditions as follows:

- The zinc finger structures could be applied for engineering proteins that target specific genes.
- Combining zinc fingers with other effector domains can enable genome manipulation [91].
- Fusion of zinc finger peptides to repression or activation domains could switch the genes off or on. It could manage conditions like genetic diseases, cancer, or viral infection.
- They could boost the expression of beneficial genes to generate advantageous characteristics in living organisms.

Technology has been developed to understand and manipulate ZNFs utilizing their specific ability to bind RNA. It has led to the development of synthetic ZNFs, which can activate, repress, or create defined changes to user-specified genes in human cells, plants, and other organisms [92]. They also enable external control of protein activity and delivery and protein and enzyme function evolution.

Another metal ion can replace the Zn in zinc fingers as an inhibition method. For instance, gold, platinum, cobalt, and selenium complexes may be developed as zinc finger inhibitors for therapy [93]. However, the main challenge in designing these inhibitors is selectivity.

## 4. Conclusion

The present review provides an insight into the realm of ZNFs, particularly their influence on conditions like oral cancer and periodontitis. As the ZNFs affect cancer development, progression and metastasis, and inflammatory pathways, they can influence the disease severity. They function as tumor suppressors or oncogenes and have anti-inflammatory effects. Drug therapy to target specific ZNFs may be utilized to avoid their expression. Owing to the high specificity in function and expression of some ZNFs in tumors, this class of proteins could be applied as prognostic markers.

## Figures and Tables

**Table 1:** Studies evaluating the role of zinc finger proteins in various oral conditions.

Author	Oral condition evaluated	ZNF studied
Codd, JD et al., 1999 [28]	Nasopharyngeal carcinoma	A20
Foster KW et al., 1999 [40]	OSCC	GKLF
Kubota T et al., 2001 [36]	Periodontitis	ZF-9
Gonzalez HE et al., 2003 [41]	OSCC	ZNF-185
Takkunen M et al., 2006 [26]	OSCC	Snail
		ZEB1
		ZEB2
Tang Y et al., 2010 [42]	Salivary adenoid cystic carcinoma	Slug
Chiang WF et al., 2011 [43]	OSCC	ZASC1
Katafiasz D et al., 2011 [24]	Squamous cell carcinoma	Slug
Wang Y et al., 2011 [44]	Oral verrucous carcinoma	ZNF77
Wang C et al., 2012 [27]	Squamous cell carcinoma of tongue	Snai2
Schaefer AS et al., 2014 [39]	Periodontitis	IRF-5
		PRDM1
Cho E et al., 2015 [45]	OSCC	REST
Ko CP et al., 2015 [46]	OSCC	MZF1
Lei W et al., 2015 [31]	OSCC	ZEB1
Li W et al., 2015 [30]	OSCC	KLF4
Ma H et al., 2015 [47]	Squamous cell carcinoma of tongue	ZFX
Ohshima J et al., 2015 [37]	Periodontitis	ZEB 2
Sekine J et al., 2015 [48]	OSCC oral epithelial dysplasia	NAC1
Cannonier SA et al., 2016 [19]	OSCC	Gli2
Cheng SJ et al., 2016 [17]	OSCC	ZNF582
Hong JY et al., 2016 [9]	Periodontitis	A20
KAmata Y et al., 2016 [23]	OSCC	Snail-1
Sztukowska MN et al., 2016 [33]	OSCC	ZEB 1
Wu B et al., 2016 [49]	OSCC	ZEB 1
Xue P et al., 2016 [56]	Periodontitis	MORF
Ding X et al., 2017 [29]	OSCC	KLF7
Huaitong X et al., 2017 [20]	OSCC	Gli1
Lee J et al., 2017 [25]	OSCC	Slug`
		Snail
		ZEB1
Maekawa S et al., 2017 [21]	Periodontitis	Gli1
Mikami Y et al., 2017 [22]	OSCC	KRT17
		Gli-1
		Gli-2
Pan L et al., 2017 [13]	OSCC	ZNF750
Wang H et al., 2017 [7]	OSCC	ZNF703
Yang H et al., 2017 [15]	OSCC	ZNF750
Yao X et al., 2017 [35]	OSCC	ZEB1
Matsui S et al., 2018 [38]	Periodontitis	ZEB 1
Cheng SJ et al. 2018 [16]	OSCC oral dysplasia	ZNF582
Kurihar-Shimomura M et al., 2018 [50]	OSCC	ZFAND4
Pan L et al., 2018 [14]	OSCC	ZNF750
Ren W et al., 2018 [32]	OSCC	ZEB2
Rodrigues MFSD et al., 2018 [51]	OSCC	Gli3
Suárez-Canto J et al., 2018 [52]	OSCC	ZFAND4
Zhao C et al., 2018 [53]	OSCC	ZNF662
Hämäläinen L et al., 2019 [54]	Oral lichen planus	ZEB1
Liu X et al., 2019 [12]	OSCC	ZNF 750
Sun R et al., 2020 [18]	OSCC	PAX1
		ZNF582
Wu J et al., 2020 [34]	OSCC	ZEB2
Yeh LY et al., 2020 [55]	OSCC	ZBTB7A
Hernández HG et al., 2021 [57]	Periodontitis	ZNF718
		HOXA4
		ZNF57

## Data Availability

All the data used to support the findings of this review are included within the article.
